# Biosynthetic Plastics as Tunable Elastic and Visible Stent with Shape‐Memory to Treat Biliary Stricture

**DOI:** 10.1002/advs.202303779

**Published:** 2023-08-08

**Authors:** Wei Wang, Zhaohui Luan, Zhenzhen Shu, Kaige Xu, Tongchuan Wang, Shuang Liu, Xiaozhuo Wu, Hangzong Liu, Shaosong Ye, Ruijue Dan, Xiaoyan Zhao, Shiming Yang, Malcolm Xing, Chaoqiang Fan

**Affiliations:** ^1^ Department of Gastroenterology Xinqiao Hospital Army Medical University NO.183, Xinqiao Street Chongqing 400037 China; ^2^ Department of Mechanical Engineering University of Manitoba Winnipeg Manitoba MB R3T 2N2 Canada; ^3^ Chongqing Municipality Clinical Research Center for Gastroenterology, Office of Science and Technology of Chongqing No. 2 Xingai road Chongqing, Yubei 401147 China; ^4^ Chongqing Institute for Brain and Intelligence, Guangyang Bay Laboratory Chongqing 400064 China

**Keywords:** biliary stricture, biodegradable stent, endoscopy, polyhydroxyalkanoate, shape memory, triamcinolone acetonide

## Abstract

Common biliary tract is ≈4 mm in diameter to deliver bile from liver to small intestine to help digestion. The abnormal narrowing leads to severe symptoms such as pain and nausea. Stents are an effective treatment. Compared with non‐degradable stents which require repeated removal, biodegradable stents have the advantage of reducing secondary injury related to endoscopic operation and patient burden. However, current biodegradable materials may cause tissue hyperplasia and the treatment method does not target etiology of stricture. So recurrence rates after biodegradable stent implantation are still high. Here, a biodegradable helical stent fabricated from biosynthetic P(3HB‐*co*‐4HB) is reported. Tunable properties can be acquired through altering culture substrates. Stent shows shape memory in various solvents. The stent has an optimized design with helical structure and outer track. The self‐expanding of helical structure and double drainage realized by outer track greatly improve drainage of bile. Importantly, stent‐loading triamcinolone acetonide can inhibit proliferation of fibroblasts and reduce incidence of restricture. Therapeutic effect is also demonstrated in minipigs with biliary stricture. The results of minipig experiments show that biliary duct in treatment group is unobstructed and tissue hyperplasia is effectively inhibited.

## Graphical abstract

A biodegradable helical stent with shape memory and self‐expanding effect is fabricated from biosynthetic P(3HB‐*co*‐4HB) with tunable stiffness for the first time. The stent successfully treats biliary stricture in porcine model.

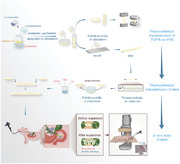

## 1 Introduction

Biliary stricture (BS) is an abnormal narrowing of the bile duct, which is usually caused by cancer, cholecystectomy, liver transplantation and chronic pancreatitis.^[^ ^1^ ^]^ BS will cause jaundice, acute cholangitis, and secondary biliary cirrhosis.^[^ ^2^ ^]^ Repeated infection in the biliary system caused by long‐term bile duct obstruction may result in biliary stones, serious liver damage and even bile duct cancer^[^ ^3^ ^]^ and its main goal is to relieve biliary obstruction and maintain normal liver function. Polyethylene stents are commonly used in the clinic. But they are not biodegradable and prone to obstruct due to their small lumen (1.65–4 mm) and nonexpandable properties. The stent patency was reported to be 77 to 126 days.^[^ ^4^ ^]^ The stents should be removed and replaced periodically every 3–4 months for 12 months.^[^ ^5^ ^]^ Besides, the removal and replacement of stents may cause potential complications such as bile duct damage and increase patients' economic and psychosomatic burden.

Biodegradable stents (BBDs) have already been widely used in cardiovascular, esophagus and trachea,^[^ ^6^ ^]^ they have emerged to avoid multiple removal and related complications. Recently, BBDs have obtained intense interest. For example, ELLA‐BD stent (ELLA‐CS) and Archimedes stent (Amg International GmbH) were developed for clinics, but there are some concerns. The recurrence rate of stricture using Ella stent can be 26.6−29.4%.^[^ ^7^ ^]^ The two stents have the following disadvantages. Firstly, they are mainly made of synthetic polymer polydioxanone (PDX). The degradation products of PDX can acidize the local environment and cause potential tissue hyperplasia reactions to the bile duct.^[^ ^8^ ^]^ Second, BBDs do not have shape memory functions. Shape memory property can endow stent self‐expanding effect, which can greatly increase the lumen diameter when the stent is inserted into the bile duct.^[^ ^9^ ^]^ A large lumen is necessary for a smooth drainage. Third, the degradation rate ranges between 11 days and 6 months,^[^ ^10^ ^]^ which does not meet the guideline‐recommended time (>12 months). Fourth, the two stents are no drug‐loading stents for biliary stricture and can't specifically treat the pathogenesis of biliary stricture.

**Scheme 1:**
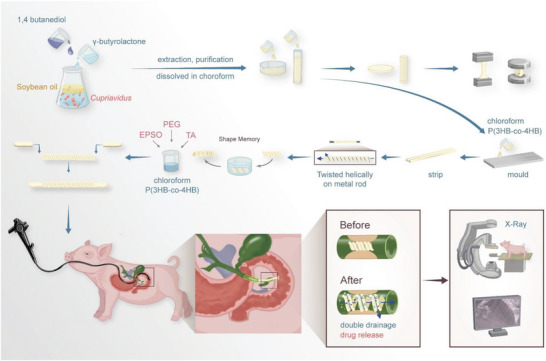
The fabrication process of stent and insertion into bile duct endoscopically for the treatment of biliary stricture. TA: Triamcinolone acetonide. PEG: Polyethylene glycol. EPSO: Ethiodized poppy seed oil.

An ideal biliary stent shall meet the following requirement: 1) It should provide sufficient mechanical support and maintain the patency of bile duct. 2) It should have a suitable degradation time, preferably a tunable degradation time, to ensure the remodeling of the bile duct before stent degradation. It can avoid the complications caused by a long stay of stent in bile duct.^[^ ^11^ ^]^ 3) It should have good biocompatibility and will not cause tissue hyperplasia. And 4) It can act as a therapeutic platform to carry drugs, not just a mechanical support role. In address these concerns, we customized *Cupriavidus necator* to acquire poly(3‐hydroxybutyrate‐*co*‐4‐hydroxybutyrate) [P(3HB‐*co*‐4HB)]. Then we designed a new shape‐memory and drug‐loading stent in our study. As a linear copolyester, P(3HB‐*co*‐4HB) is formed by copolymerization of 4‐hydroxybutyric acid (4‐HB) and 3‐hydroxybutyric acid (3‐HB). Unlike synthetic polymers such as PDX, P(3HB‐*co*‐4HB) is yielded by microorganisms under specific growth conditions.^[^ ^12^ ^]^ Their properties can be modulated by changing the substrate of microorganisms.^[^ ^13^ ^]^ By this way, we obtained P(3HB‐*co*‐4HB) with required mechanical properties related to compression, elasticity and tunable degradation rate to shape the biliary tract.^[^ ^2^ ^]^ P(3HB‐*co*‐4HB)s have good biocompatibility and cause less stimulation to tissues due to less acidic degradation products.^[^ ^14^ ^]^ This helical P(3HB‐*co*‐4HB) stent presents shape memory and self‐expanding property in different solvents. The structure was further optimized by track on the outer surface that enables the stent to drain bile internally and externally. Moreover, the delivery of triamcinolone acetonide (TA) by stent locally can suppress myofibroblast proliferation which is the primary cause of benign biliary stricture (BBS). This can alleviate the stricture recurrence rate and achieve long‐term therapeutic effect. The feasibility and safety of stents were verified in minipig model of biliary stricture (**Scheme** **1**).

## 2 Results and Discussions

### 2.1 P(3HB‐*co*‐4HB) with Diverse Properties Produced by *Cupriavidus necator*

**Figure 1:**
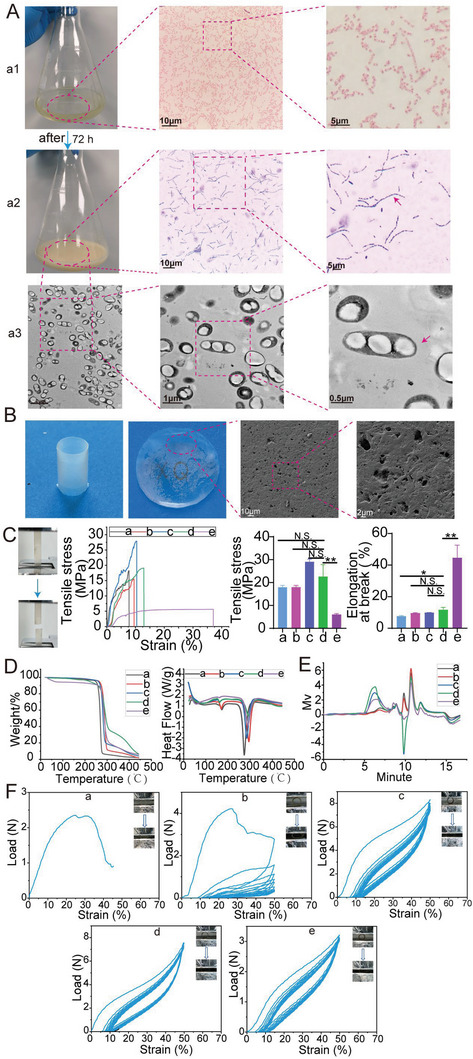
The production and physicochemical properties of P(3HB‐*co*‐4HB). A) The production process of P(3HB‐*co*‐4HB). a1) Gram staining of *Cupriavidus necator* strain; a2) Staining of intracellular P(3HB‐*co*‐4HB) with Sudan Black; a3) Observation of intracellular accumulation of P(3HB‐*co*‐4HB) with TEM; The red arrow indicating the intracellular P(3HB‐*co*‐4HB). B) Images of film and tubular stent constructed with extracted P(3HB‐*co*‐4HB) and SEM images of P(3HB‐*co*‐4HB) film. C–F) Physicochemical properties of P(3HB‐*co*‐4HB) with different monomer ratios. Five materials (a–e) were characterized by tensile tests (C), thermal gravity analyzing (D), gel permeation chromatography (E), and compression tests (F).

The intracellular accumulation of P(3HB‐*co*‐4HB) granules was observed following Sudan black staining, which was also confirmed under TEM (**Figure** **1**A). Under SEM, the surface showed porous structure with different sizes and fibers aligned in different directions (Figure 1B).

Peaks at about 2933, 2855, and 1720 cm^−1^ were characteristic peaks of P(3HB‐*co*‐4HB) which corresponded to the C─H stretching vibrations of ─CH_3_, ─CH_2_ and ester carbonyl (>C═O) respectively (Figure S1, Supporting Information).^[^ ^15^ ^]^ Absorption peaks at ≈3397cm^−1^ (O─H stretching) and 1602 cm^−1^(C═C) represented unique TA characteristic peaks.^[^ ^16^ ^]^ Polyethylene glycol (PEG) characteristic peaks located at around 2875, 1455, and 1357 cm^−1^ corresponded to C─H stretching, C─H bending vibrations and the combination of C─O─H and O─C─H deformation.^[^ ^17^ ^]^

The weight‐average molecular weight (*M*_w_), number‐average molecular weight (*M*_n_) and polydispersity index (PDI, *M*_w_/*M*_n_), tensile strength, elongation at break, melting temperature (*T*_m_), and decomposition temperature (*T*_d_) were shown in Figure 1C–F and summarized in **Table** **1**.

**Table 1:** Diverse properties of P(3HB‐*co*‐4HB) with different monomer ratios

	Type	Carbon source	3HB [%]	4HB [%]	*M*_w_ (10^3^)	*M*_n_ (10^3^)	PDI (*M*_w_/*M*_n_)	Tensile strength [MPa]	Elongation at break [%]	*T*_m_ [°C]	*T*_d_ [°C]
1	a	Soybean oil (20 g L^−1^)	100	0	76	39	1.9	17.9	7.6	172.1	273.4
2	b	Soybean oil (20 g L^−1^) + 1,4‐butanediol (5 g L^−1^)	100	0	189	59	3.2	15.9	9.5	169.7	294.2
3	c	Soybean oil (20 g L^−1^)+ γ‐butyrolactone (5 g L^−1^)	95.3	4.7	288	81	3.53	29.1	9.9	163.1	284.7
4	d	Soybean oil (20 g L^−1^)+ γ‐butyrolactone (5 g L^−1^)	93.9	6.1	502	142	3.54	20.2	11.7	162.7	293.4
5	e	Soybean oil (20 g L^−1^)+ γ‐butyrolactone (10 g L^−1^)	86.6	13.4	296	82	3.59	6.0	44.7	161.4	289.7

Note: *T*_m_: melting temperature. *T*_d_: decomposition temperature.

^1^HNMR spectroscopy showed the monomer content and chemical structure of P(3HB‐*co*‐4HB). Characteristic peaks of P3HB at *δ* = 5.11–5.26 ppm (─CH─, 2), *δ* = 2.41–2.66 ppm (─CH_2_─, 3), and 1.58 ppm (CH_3_, 1) were shown. Chemical shifts at 4.11 ppm (─CH_2_─, 4) represented 4HB. The fraction of each monomer was determined by the area at 5.26 ppm from 3HB and 4.11 ppm from 4HB (Figure S2, Supporting Information).^[^ ^18^ ^]^

These results indicated that *Cupriavidus necator* could yield P(3HB‐*co*‐4HB) with tunable properties and different monomer ratios by adjusting culture substrates. The *M*_w_ of five materials ranged between 76 and 502 kDa. And M_n_ ranged between 39 kDa and 142 kDa. Polydispersity varied between 1.9 and 3.59. There was no clear relationship between *M*_w_, *M*_n_, and 4HB content, because molecular weight lied on a number of factors such as carbon source, bacterial types, culture time, and extraction technology. With the increase of 4HB content from 4.7% to 13.4%, the tensile strength lowered from 29.1 to 6 MPa, but the elongation at break rose to 44.7% from 9.9%. The elasticity of the copolymer was improved by introducing 4HB into the copolymer, which was consistent with other researchers.^[^ ^18b^ ^]^ When 4HB was introduced into P3HB, melting point *T*_m_ decreased and decomposition temperature *T*_d_ increased. With the increase of 4HB content to 13.4%, *T*_m_ decreased from 163.1 to 161.4 °C. But there was no obvious correlation between *T*_d_ and 4HB content. *T*_d_ was influenced by molecular weight and other factors. The tubular structure made from five materials presented different compressive properties. The tubular structures made from a and b were fragile and easily compressed into fragments during compression cycling tests. The tubular structures made from c–e possessed better elasticity. But radial load declined with the increase of 4HB content. In consideration of the good compression and tensile properties of material d, we finally chose it to construct the stent. P(3HB‐*co*‐4HB) appearing in the following paper was P (93.9%3HB‐*co*‐6.1%4HB) if not stated otherwise.

### 2.2 In Vivo Biocompatibility of P(3HB‐*co*‐4HB)

**Figure 2:**
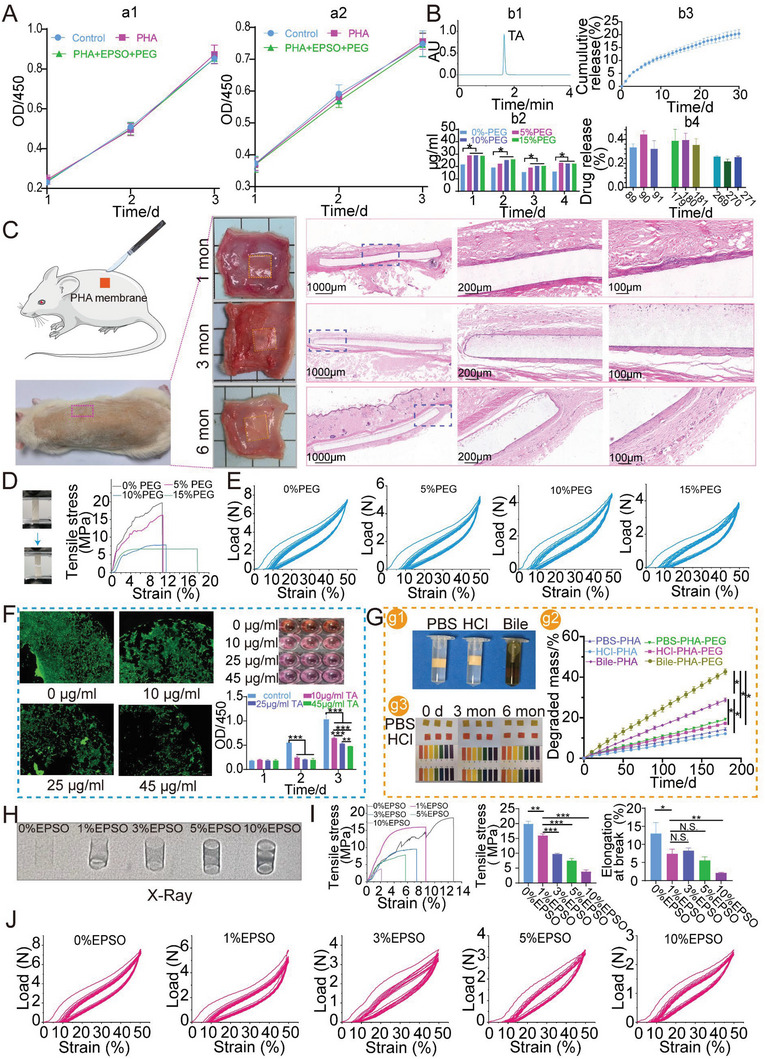
The biocompatibility of P(3HB‐*co*‐4HB), the incorporation of PEG and EPSO to increase TA release and stent visibility respectively. A) Toxicity of P(3HB‐*co*‐4HB) on 3T3 fibroblast cells (a1) and mouse extrahepatic bile duct epithelial cells (a2). PHA stands for P(3HB‐*co*‐4HB) in this figure. B) Releasing characteristics of P(3HB‐*co*‐4HB) film loading TA. b1) The HPLC curve of TA standard. b2) TA releasing curves of P(3HB‐*co*‐4HB)‐TA films with different contents of PEG. b3) TA releasing curves in the first month. b4) TA releasing curves at 3, 6, and 9 months. C) Biocompatibility of P(3HB‐*co*‐4HB) in rats. D) Tensile properties of P(3HB‐*co*‐4HB) films with different contents of PEG. E) Compressive properties of P(3HB‐*co*‐4HB) stents with different contents of PEG. F) Inhibition against 3T3 cells of different concentrations of TA in vitro. G) Degradation profiles of P(3HB‐*co*‐4HB) films in PBS, HCl and bile in vitro. g1) Images of three groups. g2) pH changes during degradation of P(3HB‐*co*‐4HB). g3) Degradation curve of P(3HB‐*co*‐4HB) films. H) Visibility of P(3HB‐*co*‐4HB) stents with different contents of EPSO under X‐ray. I) Tensile properties of P(3HB‐*co*‐4HB) with different contents of EPSO. J) Compression properties of P(3HB‐*co*‐4HB) stents with different contents of EPSO.

The cytotoxicity of P(3HB‐*co*‐4HB) was evaluated according to CCK‐8 assay. 3T3 fibroblast cells and mouse extrahepatic bile duct epithelial cells were used. The results showed no significant difference among control group, P(3HB‐*co*‐4HB) group and P(3HB‐*co*‐4HB) group with EPSO and PEG (**Figure** **2**A). The results indicated that P(3HB‐*co*‐4HB) possessed good cell biocompatibility on both cells. So it could be inferred that P(3HB‐*co*‐4HB) could be applied in various biomedical fields, which was also in accordance with other researchers.^[^ ^19^ ^]^

From Figure 2C, we could see tissue encapsulation formed after P(3HB‐*co*‐4HB) film was placed subcutaneously. After 1 month, the implanted film elicited a slight tissue response with infiltration of a few lymphocytes, neutrophils, eosinophils, and macrophages. After 3 months, the number of inflammatory cells around P(3HB‐*co*‐4HB) film decreased while fibroblast cells increased in number. The direction of tissue alignment was in parallel with the interface indicating the reduction of tissue response.^[^ ^20^ ^]^ After 6 months, tissue response decreased to be very mild and there were only few inflammatory cells. The above results showed that P(3HB‐*co*‐4HB) presented mild tissue response and had good biocompatibility in rat.

### 2.3 Drug Release Could be Increased by Incorporating PEG and Its In Vitro Profiles

An ideal stent would not only play the role of mechanical support, but also serve as a therapeutic platform, which could carry drugs and other therapeutic media.^[^ ^21^ ^]^ Drug‐eluting stents have been widely used in the cardiovascular field to reduce intimal hyperplasia.^[^ ^22^ ^]^ The application of drug‐loaded stents in biliary stents was still rare. Anti‐cancer drugs such as paclitaxel were usually used as therapeutic drugs to inhibit bile duct tissue hyperplasia.^[^ ^23^ ^]^ TA was a commonly used glucocorticoid with anti‐inflammatory sol effects in clinics. Glucocorticoids had previously been used to treat scars^[^ ^24^ ^]^ and recurrent or refractory benign esophageal stricture.^[^ ^25^ ^]^ Local release of TA had achieved good results in the prevention and treatment of benign biliary stricture. But they either used surgical methods to implant therapeutic medium or drug release lasted for a short time in previous studies.^[^ ^26^ ^]^ Therefore, it was promising to achieve long‐term local drug release by drug‐loading stents which was minimally invasive. The mobility of bile in the biliary duct posed a challenge to drug release. TA was a lipophilic drug that could bind closely with tissues and avoid being washed away by bile. By local release, the systemic side effects of the drug could be reduced and the local concentration of the drug could also be increased to the enhance treatment effect.^[^ ^27^ ^]^ Therefore, we tried to load TA on the stent in this study.

Polyethylene glycol (PEG) was a good compatibilizer which was often employed as a regulator in drug release.^[^ ^28^ ^]^ PEG could increase the hydrophilicity of the system. As PEG could be released rapidly, voids or pores would generate and the later drug release speeded up. We could see that the release of the drug was improved by adding PEG and the improvement of 5% PEG, 10% PEG and 15% PEG on the release of the drug was similar (Figure 2B‐b1,b2). The tensile tests showed that the elongation at break increased with PEG concentration, but the tensile strength decreased inversely (Figure 2D). When the PEG content increased to 10%, the elongation at break increased, but the tensile strength decreased largely. As shown in the compression tests, with the increase of PEG, the supporting force gradually decreased (Figure 2E). In consideration of the above results, 5% PEG was finally selected.

We further studied whether the released amount of TA could inhibit 3T3 cells. The solubility of TA in water was about 45 µg mL (Figure S3A, Supporting Information). Four concentrations of TA (0, 10, 25, and 45 µg mL^−1^) were used. TA could inhibit the growth of the cells when TA's concentration was over 10 µg mL^−1^ (Figure 2F). In comparison with the control group, released TA of the above PEG drug‐loaded films could reach the inhibitory concentration of TA on 3T3 cells which was consistent with other literature (Figure S3B, Supporting Information).^[^ ^29^ ^]^ The drug release profile of 5% PEG‐P(3HB‐*co*‐4HB) film loading TA was further investigated (Figure 2B‐b3,b4). We found that although the drug release showed a downward trend, the released TA could still be detected at the end of 9 months.

### 2.4 In Vitro Degradation of P(3HB‐*co*‐4HB)

In vitro degradation profile of P(3HB‐*co*‐4HB) was studied in PBS, HCl, and bile (Figure 2G‐g1). From Figure 2G‐g2, it could be seen that the PEG group degraded faster than 0% PEG‐P(3HB‐*co*‐4HB) group. Under same conditions, the degradation rate in bile was faster than that in PBS and HCl, which was due to the components in bile^[^ ^30^ ^]^ and the degradation rate in PBS was faster than in HCl. According to the fitting formula obtained from the degradation data of 6 months, the time for complete degradation of P(3HB‐*co*‐4HB) in PBS, bile, and HCl was estimated to be 1323.0, 631.9, and 1516.3 days and the degradation time of 5%PEG‐P(3HB‐*co*‐4HB) in PBS, bile, and HCl was estimated to be 977.7, 424.6, and 1082.9 days respectively. Compared with PDX,^[^ ^31^ ^]^ the degradation time of P(3HB‐*co*‐4HB) was significantly prolonged, demonstrating that the mechanical properties of stents constructed by P(3HB‐*co*‐4HB) could be maintained longer and it would benefit the remodeling of biliary tract and reduce the rate of restricture. As from Figure 2G‐g3, the pH of PBS and HCl media did not change obviously after 3 and 6 months. So it could be inferred that degradation process of P(3HB‐*co*‐4HB) had little influence on local pH, which could be ascribed to its slow degradation rate and its low acidity of degradation products.^[^ ^14a^ ^]^ PDX had an evident impact on pH. When PDX suture was placed in buffer for 10 weeks, the pH of the solution dropped from 7.4 to 5.6.^[^ ^32^ ^]^ The decrease of local pH would stimulate tissue proliferation. So it could be inferred tissue hyperplasia caused by P(3HB‐*co*‐4HB) stent might be improved.

### 2.5 EPSO Added to Increase Stent Visibility

Visibility is an essential property of stents for the proper placement and localization. In the past, the visibility of biodegradable stents was generally realized by adding contrast agents such as barium sulfate.^[^ ^33^ ^]^However, barium sulfate was solid and not easily soluble in chloroform. As the stent degraded, the barium sulfate released might absorb water and form gallstones to block the biliary tract.^[^ ^34^ ^]^ In our study, ethiodized poppy seed oil (EPSO), a common oily contrast agent in clinics,^[^ ^35^ ^]^ was used to enhance the visibility of stent. EPSO was a safe liposoluble contrast agent. It could be absorbed by tissue and would not form gallstones in bile duct. Tubular stents with five concentrations of EPSO (0%, 1%, 3%, 5%, and 10%) were compared. Under X‐ray, with the increase of iodine content, the visibility of the stent was gradually enhanced (Figure 2H). The tensile strength and elongation were down with the increase of EPSO concentration (Figure 2I). Compression tests showed that the support decreased when EPSO concentration increased (Figure 2J). The stent of 1% EPSO presented good visibility and maintained a similar mechanics. Thus, this group was selected as the final product of this study.

### 2.6 The Characterization of Stent

**Figure 3:**
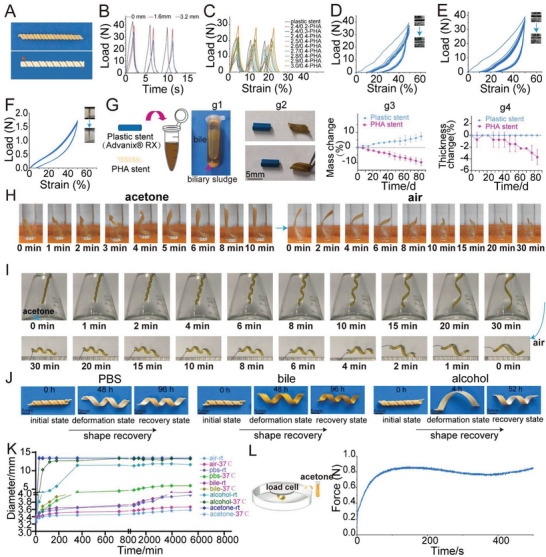
Characterization and shape memory behaviors of P(3HB‐*co*‐4HB) helical stent. A) The photograph of P(3HB‐*co*‐4HB) helical stent with outer track. B) The cyclic compression performance of P(3HB‐*co*‐4HB) helical stent with different pitches (strain 25%). C) The cyclic compression performance of plastic stent and P(3HB‐*co*‐4HB) helical stent with different thicknesses and diameters (strain 25%). PHA stands for P(3HB‐*co*‐4HB) in this figure. D) The cyclic compression performance of plastic stent (strain 50%). E) The cyclic compression performance of P(3HB‐*co*‐4HB) stent (2.8 mm in diameter, 0.4 mm in thickness, strain 50%). F) The longitudinal cyclic tensile performance of P(3HB‐*co*‐4HB) helical stent (2.8 mm in diameter, 0.4 mm in thickness). G) Adhesive properties of P(3HB‐*co*‐4HB) helical stents and polyethylene plastic stents against biliary sludge in vitro. g1) Aged bile with biliary sludge. g2) Images of plastic stent and P(3HB‐*co*‐4HB) stent immersed in bile for 3 months. g3) Mass changes of two stents at different time points. g4) Thickness changes of two stents at different times. H) Shape change of P(3HB‐*co*‐4HB) helical stent before and after immersion in acetone at room temperature. I) Shape change of P(3HB‐*co*‐4HB) helical stent when hanging in acetone bottle and being taken out. J) The shape recovery of P(3HB‐*co*‐4HB) stent in 37 °C PBS, bile and alcohol at different time points. K) Self‐expansion of P(3HB‐*co*‐4HB) helical stent in air, PBS, bile, alcohol, and acetone at room temperature (rt) and 37 °C. L) Expansion force of P(3HB‐*co*‐4HB) helical stent in acetone at different times.

Compared with other lumens, the biliary tract (6–8 mm in human) was smaller and easier to block, so the drainage of biliary stent was critical to the treatment of biliary stricture. Otherwise, the accumulated bile would cause biliary severe infections. We combined helical and tubular structures with teeth in the stents. The helical structure presented good mechanical properties and shape memory (**Figure** **3**A).^[^ ^36^ ^]^ The tubular structure with teeth could prevent migration and its small lumen could reduce the reflux of intestinal contents, which would cause stent failure.

As shown in the Figure 3B, the supporting force of the stent gradually decreased as the pitch (distance between two coils) increased from 0 to 1.6 mm, 3.2 mm. In consideration of the good supporting force, we finally chose the type of 0 mm. The supporting force of the stent gradually increased with the coil thickness increasing from 0.2 to 0.4 mm and diameter from 2.4 to 3.0 mm. We compared the plastic stent Advanix made of polyethylene with our P3HB‐P4HB stents at 25% deformation (Figure 3C and Figure S4, Supporting Information). The maximum support of this commercial stent (2.4 mm in diameter) was about 21.8 N which was comparable with 19.7 N in 2.7 mm (diameter)/0.4 mm(thickness), 20.3 N in 2.8 mm/0.4 mm and 22 N in 2.9 mm/0.4 mm of P(3HB‐*co*‐4HB) stent. Considering the limited inner diameter of working channel in endoscope, we finally chose the 2.8 mm/0.4 mm for the helical stent. The cyclic compression characteristics of the commercial stent and P(3HB‐*co*‐4HB) stent (2.8/0.4) at 50% deformation were shown in Figure 3D,E (Video S1, Supporting Information). The longitudinal cyclic tensile tests of 50% strain showed that the stent had a good recovery ability (Figure 3F). When the stent was longitudinally deformed under external force after placement in the biliary tract, it could go back to its original shape. This was important for the mechanical support requirement.

Bile contained calcium palmitate and bilirubin, which gave the potential to form gallstones.^[^ ^37^ ^]^ Normal bile would gradually form biliary sludge consisting of gallstones after standing in vitro. Biliary sludge was the main reason for occluded stent and causing restricture.^[^ ^38^ ^]^ So it is a crucial characteristic determining whether the stent is easy to adhere to bile sludge. We used aged bile, because it had more bile sludge and was easy for us to observe the adhesion (Figure 3G‐g1,2). As time went on, we observed that the mass of P(3HB‐*co*‐4HB) stent gradually decreased, while the commercial stent showed an upward trend (Figure 3G‐g3). The thickness of the commercial stent did not change significantly and the biliary mud would gradually adhere to the surface of plastic stent, which might cause the stent obstruction. In contrast, P(3HB‐*co*‐4HB) stent showed a downward trend (Figure 3G‐g4) and a self‐cleaning effect against bile sludge, because the biliary sludge adhering to the surface would gradually fall off with the degradation of the stent.^[^ ^39^ ^]^ This self‐cleaning effect of P(3HB‐*co*‐4HB) stent would maintain long‐term patency of stent.

### 2.7 Shape Memory and Self‐Expanding Test

Shape memory in stent has received an intense interest.^[^ ^40^ ^]^ The stent presents delivering potential via endoscope system in a compressibly squeezed form. With shape memory, upon stimuli, the stent can recover its original shape to expand the narrowed duct. Currently most BBDs do not have this function. Previous studies showed good shape memory by copolymerizing PHA with other materials such as polyurethane.^[^ ^9^, ^41^ ^]^ We observed that our P(3HB‐*co*‐4HB) also presented memory effect in different solvents. Memory effect of P(3HB‐*co*‐4HB) in acetone and alcohol was most significant and also obvious in PBS and bile. We investigated the shape memory in acetone (Figure 3H and Video S2, Supporting Information). When stent was immersed in acetone at room temperature, it was gradually unfolded. After 10 min, when no further deformation happened, the stent was moved to a glass tube. The stent gradually returned to its original shape and the diameter of the stent recovered to 4.2 mm at 30 min. A similar shape memory effect was observed when the stent was suspended in a conical flask containing acetone (Figure 3I and Video S3, Supporting Information). The shape memory of stent in alcohol, water and PBS was also good. (Figure 3J).

The shape memory P(3HB‐*co*‐4HB) was attributed to the interaction between solvent and polymer.^[^ ^42^ ^]^ After the addition of solvent, the hydrogen bond formed between solvent and polymer broke the original chemical structure of polymer and caused it to deform. After the solvent volatilized, the original chemical structure of polymer was restored, which was shown as shape restoration in macro.^[^ ^43^ ^]^ The difference of shape memory effect caused by different solvents was related to the permeability of solvents and the strength of hydrogen bonds formed. The presence of polar and non‐polar bonds in acetone and ethanol made it easy to interact with the polymer.^[^ ^44^ ^]^

Figure 3K showed the self‐expanding effect of P(3HB‐*co*‐4HB) helical stent under different solvents and temperature. Of the four solvents, the self‐expanding effect of the stent in acetone was the most obvious. At room temperature, the stent in PBS and bile showed almost no expansion, while temperature increased to 37 °C, the self‐expanding effect of the stent was obvious and faster. So water or solvents and temperatures both played a crucial part in the expansion. Compared to some existing biodegradable stents such as Archimedes stents, our self‐expanding stents were efficient to treat biliary stricture.^[^ ^10b^ ^]^ For the reason that the self‐expanding effect could greatly enhance the drainage effect of stent, the bile could be drained more smoothly. When inserted into bile duct, the lumen diameter would be increased largely by stent. Then we measured the self‐expanding force of the stents in acetone over time using a universal testing machine. As could be seen from the Figure 3L, when acetone was added, the force measured by the sensor gradually increased to around 0.8 N due to the expansion of the stent and reached a steady state.

### 2.8 The P(3HB‐*co*‐4HB) Helical Stent (Without Tooth) Performed Well in the Normal Bile Duct in Minipig

**Figure 4:**
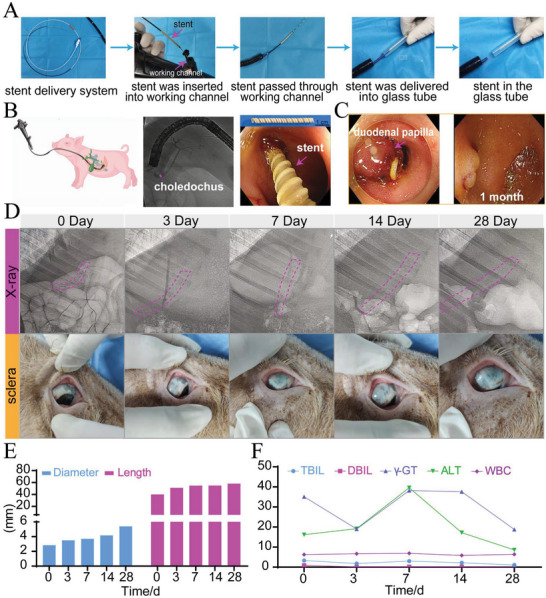
Stent without tooth insertion in vitro and in minipigs with normal bile duct. A) Endoscopic release of P(3HB‐*co*‐4HB) helical stent in vitro. B) Observation of normal bile duct in minipig through ERCP and the implantation process of stent without tooth. C) Endoscopy images of stent at day 0 and 28. D) The images of P(3HB‐*co*‐4HB) stent under X‐ray and sclera color at different times. E) Changes in diameter and length of P(3HB‐*co*‐4HB) helical stent in vivo. F) Changes of blood indexes in minipig at different times.

As a proof of concept, the helical stent was delivered into a glass tube to show the stent could be released smoothly under endoscope (**Figure** **4**A and Video S4, Supporting Information). The vital signs of minipig in the stent group (no.2 minipig) was stable during the operation (Figure S5A, Supporting Information). Endoscopic retrograde cholangiography (ERCP) showed the maximum diameter of normal bile duct measured was 3.4 mm (Figure 4B). A non‐drug‐loaded stent with a 2.8 mm in outer diameter and 40 mm in length was selected. This stent was successfully inserted into the bile duct (Figure 4B and Video S5, Supporting Information). The outer track of the stent could be clearly seen under endoscope with the stent end coming out of the duodenal papilla (Figure 4B,C). During the subsequent 1‐month follow‐up, the animal status and diet were normal. The position and expansion of the stent were observed by X‐ray at day 0, 3, 7, 14, 28 (Figure S5B, Supporting Information). We found that the stents' contour was clearly visible. No breakage occurred under X‐ray (Figure 4D). From day 3, the stent expanded onward with an increase in length. The color of minipig sclera was observed at predetermined time points. Yellow staining of the sclera did not appear (Figure 4D). After 4 weeks, the stent expanded to 5.2 mm in diameter and extended to 58 mm in length (Figure 4E).Serological results showed that total bilirubin (TBIL), direct bilirubin DBIL, alanine aminotransferase (ALT), γ‐glutamyl transferase (γ‐GT), aspartate aminotransferase (AST), lactic dehydrogenase (LDH), neutrophilic granulocyte percentage (NEUT%) and white blood cell (WBC) had no significant change (Figure 4F and Figure S7A, Supporting Information). Procalcitonin and some inflammatory factors (IL‐6, IL‐8, TNF‐α) were in the normal range during postoperation, indicating that there was no obvious inflammation (Figure S6, Supporting Information). After 4 weeks of implantation, the end of the stent could not be seen under endoscope but the stent could still be observed under X‐ray, indicating that the stent had migrated into the inner bile duct slightly (Figure 4C,D). It suggests that the stent needed further improvement.

**Figure 5:**
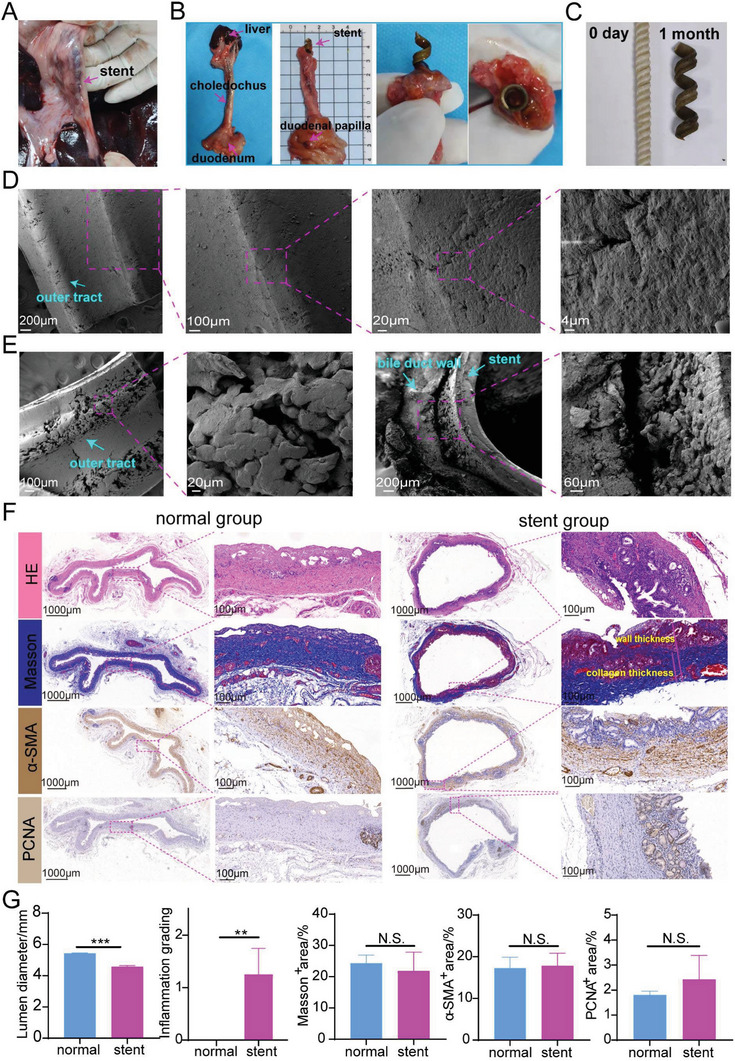
Gross and microscopic view of stents after dissection and histologic staining of the bile duct in a normal minipig. A) Position of common bile duct with a stent (without tooth) in the minipig. B) The dissected bile duct in stent group. C) The images of the initial stent and stent removed from the minipig. D) SEM images of the initial stent. E) SEM images of stents and bile duct from the minipig. F) Histologic staining of biliary tissue in normal group and stent (without tooth) group. G) Quantification analysis of lumen diameter, inflammation grading, Masson^+^ area, α‐SMA^+^ area, and PCNA^+^ area.

The two minipigs (one with stent, the other without stent) were euthanized after stent was implanted for 1 month. Outline of the stent could be seen after entering the abdominal cavity which indicated that stent was located in common bile duct (**Figure** **5**A). Gross inspection of the extrahepatic bile ducts showed no perforation. The duodenal papilla could be found along the lower segment of the stent (Figure 5B). The bile duct at the upper end of the stent was cut horizontally and bile duct showed patent indicating the stent played a good supporting function. After being taken out, the stent expanded significantly compared with the original size (Figure 5C). The biliary stent before and after implantation were then examined under SEM (Figure 5D). When inserted into bile duct for 1 month, there were cracks on the surface due to the degradation of bile (Figure 5E). Tissues including bile duct stent were observed under SEM (Figure 5E). The external surface of the stent was in close contact with the inner biliary wall and the expansion of bile duct show the good mechanical maintaining.

There were no ulcers, perforation, and tissue necrosis from hematoxylin & eosin (H&E) staining of the biliary duct (Figure 5F). Compared with the normal group (no.1 minipig without stent inserted), there was only slight inflammation in the stent group (Figure 5F,G). The two groups presented no significant difference in Masson staining and α‐SMA immunohistochemical staining. Though there was significantly different in inner diameter between the stent group (4.6 mm, no tooth) and the normal group (5.4 mm), the lumen diameters were both in normal range (Figure 5F,G). No significant difference was found in the PCNA^+^ area between two. But it could be seen that there was an upward trend in the stent group for PCNA^+^ area, suggesting that the compression of the stent stimulated mucosal hyperplasia (Figure 5F,G). Masson staining showed that collagen fibers in the submucosa of both groups were arranged neatly and there was no statistical difference in Masson^+^ area between the two groups, suggesting that the content of collagen fibers in the stent group did not increase significantly (Figure 5F,G). α‐SMA was a marker indicating differentiation of fibroblasts into myofibroblasts. Myofibroblasts could secrete collagen fibers and contract, causing scar contracture. Compared with control group, no significant difference existed in α‐SMA^+^ region in stent group (Figure 5F,G), indicating that there was no obvious proliferation of myofibroblasts in the stent group. These results indicated that the stent itself had good biocompatibility. Although they could stimulate slight mucosal hyperplasia, they would not stimulate the proliferation of fibroblasts and the deposition of collagen fibers.

### 2.9 The P(3HB‐*co*‐4HB) Helical Stent with Anti‐Migration Tooth for the Treatment of Biliary Stricture in Modeled Minipig

**Figure 6:**
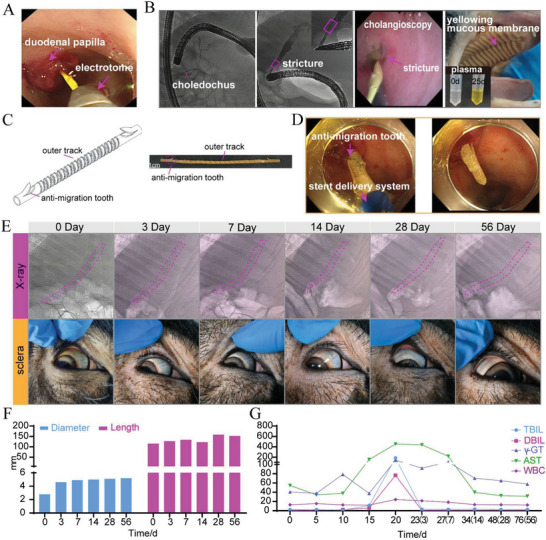
Biliary stricture model, stent insertion and therapeutic evaluation. A) Modeling of biliary stricture by cauterizing the bile duct through electrotome. B) Success of biliary stricture model was evaluated by ERCP and cholangioscopy. After successful modeling, the minipig appeared jaundice and serum color became significantly yellow. C) A P(3HB‐*co*‐4HB) stent with anti‐migration tooth. D) Stent was placed endoscopically. E) Changes of the stent and scleral colors under X‐ray at different times. The dotted red line marks the position of stent. F) Changes of stent diameter and length at different times. G) Changes of blood indexes of minipig at different times. Days after stent placement were shown in parentheses.

The good self‐expanding effect of the stent without drug was first verified in the stent group. However, the stent (without teeth) in the above stent group migrated under endoscopy after 1 month which may impair the therapeutic function. Then we considered to design anti‐migration tooth stent with drug loaded. BBS was established endoscopically in the model group (no. 3 minipig) and TA‐stent group (no.4 minipig) by thermal injury (**Figure** **6**A). Blood sample tests showed TBIL, DBIL, γ‐GT, ALT, AST, LDH, NEUT%, and WBC gradually increased (Figure 6G and Figure S7B, Supporting Information). After 20 days, TBIL increased up to184.9 µmol L^−1^ and the serum obtained after blood centrifugation could be seen to be significantly yellowed. The sclera and oral mucosa of minipig were observed significantly yellow. ERCP showed that severe stricture formed at the site 1.5–3 cm away from the duodenal papilla and bile duct was significantly expanded at the upper end of the stricture (Figure 6B). The successful modeling was further verified through direct observation under choledochoscope (Figure 6B, Video S6, Supporting Information). The drug‐loaded biliary stent (TA‐stent group) with an outer diameter in 2.8 mm and a length in 11.5 cm ensuring that the stent crossed the narrow area was made and inserted successfully (Figure 6C,D and Video S7, Supporting Information).

The vital signs of both minipigs were stable all through the operation. During the follow‐up period of 2 months, the animal diet was normal (Video S8, Supporting Information) and the position and expansion of the stent were observed by X‐ray at day 0, 3, 7, 14, 28, and 56. From X‐ray images (Figure 6E), the outline of the stent was clear with teeth at both ends. The main helical structure of stent expanded from day 3 with the increase of length. The stent expanded to 5.3 mm at day 56 (Figure 6F). After 3 days, the sclera of minipig returned to white from yellow (Figure 6E). And TBIL, AST and WBC decreased significantly. With the relief of bile duct obstruction, previously deposited bile was drained through the ducts into the intestine. Infection indicators such as WBC also gradually decreased to normal after 14 days (Figure 6G).

**Figure 7:**
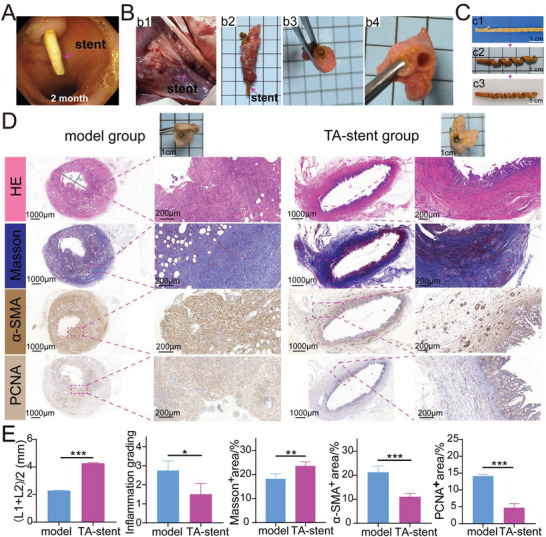
Gross and microscopic view of stents after dissection and histologic staining of the bile duct in modeled minipigs. A) Observation of stent under endoscope after insertion of 2 months. B) The position of the common bile duct and the stent. It could be seen the bile duct and stent was unobstructed. C) Shape memory characteristics of the stent at different times. c1) The initial stent (IS). c2) Fresh stent just removed from biliary duct (FS). c3) The dried stent (DS) removed from biliary duct. D) Histologic staining of bile duct in two group. L1: The longest distance in lumen, L2: The shortest distance in lumen, (L1+L2)/2 was used to estimate the lumen diameter because the lumen is irregular. E) Quantification analysis of lumen diameter, inflammation grading, Masson^+^ area, α‐SMA^+^ area and PCNA^+^ area.

After 56 days, the stent could still be seen endoscopically (**Figure** **7**A). The outline of the stent was found (Figure 7B). There was no perforation on the extrahepatic bile ducts. The bile duct has changed to unobstructed (Figure 7B). In order to investigate the shape‐memory effects, the dried stents (DS) were compared with stents freshly (FS) removed from the minipig biliary tract and the initial stent (IS) before implantation (Figure 7C). We found that the DS recovered to 66.7% of IS.

In TA‐stent group (no. 4 minipig), H&E staining did not present any ulcer, perforation or tissue necrosis (Figure 7D). The bile duct wall of the model group (no. 3 minipig) was significantly thickened, with the largest diameter up to 3.2 mm. The mucosal tissue was severely damaged. A large number of inflammatory cells infiltrated into the bile duct wall. Plenty of collagen fibers were deposited. The fibers were arranged disordered and became nodular in dense area. A large number of myofibroblasts were seen between the fibers and the proportion of α‐SMA^+^ area was significantly increased, suggesting that there were a large number of myofibroblasts in the wall (Figure 7D,E). PCNA immunohistochemical staining also indicated that the bile duct wall was in a state of obvious hyperplasia (Figure 7D,E). Compared with the model group with lumen diameter of 2.29 mm, the diameter of the TA‐stent group was recovered well to 4.27 mm after treatment (Figure 7D,E). Inflammation was improved significantly after treatment. Compared with model group, the Masson^+^ area increased in TA‐stent group (Figure 7D,E). Because model group had significant tissue hyperplasia. The evident proliferation of fibroblasts and infiltration of a great many inflammatory cells resulted in a relatively lower proportion of collagen area in TA‐stent group. The proportion of myofibroblasts was down from 21.3% to 11.1% and tissue hyperplasia (PCNA^+^ area) as well from 14.1% to 4.7% (Figure 7D,E). These results suggested that the released TA had anti‐inflammatory effects and reduced collagen deposition by inhibiting fibroblast proliferation. The bile duct tissue had mild inflammation in TA‐stent group after 8 weeks (Figure 7D,E). Considering that there was significant infection in the bile duct prior to stent placement and WBC went as high as 24.18 × 10^9^ L^−1^, it was normal that the stent group showed mild inflammation. As reported, paclitaxel‐eluting covered metallic stents showed moderate inflammation after 6 weeks when inserted into a canine normal bile duct.^[^ ^45^ ^]^ Although TA could suppress immunity, WBC gradually decreased to normal after 2 weeks of stent implantation. This indicated that TA loading does not aggravate bile duct infection and stent had good biocompatibility.

## 3 Conclusion

By modulating culture substrate, P(3HB‐*co*‐4HB) with tunable mechanical and physicochemical properties could be produced. Stents constructed with P(3HB‐*co*‐4HB) had tunable elasticity and shape memory function. They presented good visibility under X‐ray by adding ethiodized poppy seed oil. Their structure design was characterized by helical structure with outer track and tubular structure with anti‐migration tooth at two ends. Compared with traditional stents, this stent had dual therapeutic effects. On the one hand, the self‐expansion of the main helical structure could greatly improve the drainage effect of bile. On the other hand, the anti‐inflammatory effects of stent‐released TA could target the etiology of stricture. Safety and feasibility of this stent were verified in minipigs with biliary stricture.

## 4 Experimental Section

### Materials

*Cupriavidus necator* (ATCC 17699) was bought from Biofeng Biotechnology Co., Ltd (Shanghai, China). γ‐butyrolactone(*M*_w_ = 120.104), TA, soybean oil were purchased Aladin Biotechnology Co., Ltd (Shanghai, China). Cell Counting Kit‐8 was bought from Bioground Biotechnology Co. LTD (Chongqing, China). 1,4‐butanediol(*M*_w_ = 90.12), PEG (*M*_w_ = 20 000), Cell Viability Assay Kit*Green Fluorescence, anti‐PCNA mouse monoclonal antibody and Sudan black B, were purchased from Sangon Biotechnology Co., LTD (Shanghai, China), EPSO (iodine content, 480 mg mL^−1^)was purchased from Jiangsu Hengrui Pharmaceutical Co. LTD (Lianyungang, Jiangsu). Plastic stents (Advanix, Boston Scientific International LTD) were purchased from Boston Scientific International LTD. Anti‐α‐SMA mouse monoclonal antibody was purchased from Sanying (Wuhan, China). 3T3 fibroblasts cells were bought from Cell bank of the Chinese Academy of Sciences. Mouse extrahepatic bile duct epithelial cells were purchased from iCell Bioscience Inc. All other chemicals in this experiment were of analytical grade from local suppliers.

### Culture in Erlenmeyer Flasks

The nutrient broth (NB), mineral salt medium (MSM), and trace element solution was prepared according to the method of Obruka.^[^ ^46^ ^]^ Oil, MSM, microelement solutions, γ‐butyrolactone, and 1,4‐butanediol were autoclaved severally (121 °C, 25 min) and mixed at ambient temperature aseptically before inoculation. γ‐butyrolactone or 1,4‐butanediol were added as 4‐HB precursor. Soybean oil was added in 20 g L^−1^. The pH of solution was adjusted to 7.0 using 1 mol L^−1^ NaOH/HCl. Single colonies grew in NB medium for 24 h in orbital shaker at 200 rpm after being picked from agar plates. Then MSM inoculated with above culture solution (v/v 1%) were contained in Erlenmeyer flasks (v/v 5%) and the flasks were placed on a constant temperature shaker at 30 °C, 200 rpm. After 72 h, cells were centrifuged at 5000 rpm for 20 min and the collected cells were rinsed with distilled water. Sodium hypochlorite (active chlorine 8–12%)were added to digest for 90 min. The mixed ethanol and acetone solution (v/v 2:1) was added for precipitation after centrifugation. The yielded P(3HB‐*co*‐4HB) polymer was purified by dissolving in chloroform and centrifugation repeatedly.

In this study, five materials (a–e) with different ratios of 3HB and 4HB were produced. Material (a) was produced with only soybean oil (20 g L^−1^). Material (b) was produced with soybean oil (20 g L^−1^) and 1,4‐butanediol (5 g L^−1^) which were added to the medium simultaneously. Material (c) was produced using soybean oil (20 g L^−1^) and γ‐butyrolactone (5 g L^−1^). And γ‐butyrolactone were added 24 h after the addition of soybean oil. Material (d) was produced using soybean oil (20 g L^−1^) and γ‐butyrolactone (5 g L^−1^) which were added to the medium simultaneously. Material (e) was produced with soybean oil (20 g L^−1^) and γ‐butyrolactone (10 g L^−1^). And γ‐butyrolactone were added 24 h after the addition of soybean oil.

### Gram Stain

Experiment was carried out following instructions. Briefly, bacterial solution was smeared, dried, and fixed. Then crystal violet was used to dye the smear for 1 min and removed with tap water. Iodine solution was added for 1 min and removed under tap water. Non‐specific crystal violet staining was decolorized with anhydrous alcohol. The smear was then stained with Gram Safranin for 20–60 s and then eliminated with tap water. Then the slide was drained with absorbent paper and observed under oil lens.

### Transmission Electron Microscopy

Cells were harvested and polymer granules was observed under an electron microscope.

### Scanning Electron Microscopy

Films of P(3HB‐*co*‐4HB) were prepared by casting chloroform solution (5% w/v) on glass dish and dried at ambient temperature for 2–3 days in oven to constant weight. The morphology of film surface was observed under SEM (Crossbeam 340, Zeiss).

### P(3HB‐*co*‐4HB) characterization

Nuclear magnetic resonance (^1^H NMR) spectra were analyzed on Agilent DD2 at 600 MHz with chloroform‐d as solvent.^[^ ^47^ ^]^

Fourier transform infrared (FTIR) spectra were analyzed in the range of 450 to 4000 cm^−1^ on a Perkin–Elmer FTIR spectrometer (Spectrum TWO, PerkinElmer).^[^ ^48^ ^]^

Gel permeation chromatography was analyzed on a Waters 1515 instrument (Waters, U.S.A) equipped with a guard column MIXED 7.5 × 50 mm PL column and two MIXED‐C 7.5 × 300 mm columns and a differential refractive index detector. Chloroform was used as eluent (flow rate: 1 mL min^−1^) at 35 °C. The injection volumes and sample concentrations were 20 µL and 2 mg mL^−1^ respectively. *M*_w_, *M*_n_, and PDI were calculated.

Thermal gravimetry analysis (TGA) was performed on TGA instrument (SDT650, Waters, U.S.A) to determine the thermal characteristics of synthesized P(3HB‐*co*‐4HB). Samples (5–10 mg) were heated from ambient temperature to 450 °C at a speed of 10 °C min^−1^ under nitrogen flow (50 mL min^−1^).

Films of P(3HB‐*co*‐4HB) were prepared by pouring chloroform solution (12% w/v) on glass dish with diameter of 40 mm and dried in oven to constant weight. P(3HB‐*co*‐4HB) (12% m/v) was dissolved in chloroform and stirred thoroughly. The solution was decanted into a glass tube with 8 mm in diameter, evaporated naturally, and dried in an oven to obtain a tubular structure.

Tensile, compression, and compression cycling tests were conducted through a universal testing machine (Sanfeng, Jiangsu) with 100 and 1000 N load cell respectively.^[^ ^49^ ^]^ Each sample was tested in triplicate and the average value was presented.

### Sterilization

All samples for in vivo experiment were sterilized by hydrogen peroxide low‐temperature plasma (CDMJ‐100, Hangzhou Sanyuan Medical Co., LTD). Briefly, sample was placed in a ziploc bag and then was put in a hydrogen peroxide low‐temperature plasma sterilizer after sealing. After preheating to 45 °C, the machine continued disinfection process for 50 min.

### Cytotoxicity

3T3 fibroblasts cells and mouse extrahepatic bile duct epithelial cells were applied to evaluate the cytotoxicity of P(3HB‐*co*‐4HB).^[^ ^50^ ^]^ The sterilized P(3HB‐*co*‐4HB) film with or without EPSO and PEG were incubated in fresh medium at 37 °C for 24 h. Then materials were taken and extraction medium was acquired. 3T3 fibroblasts cells and mouse extrahepatic bile duct epithelial cells were inoculated in sterilized 96‐well plates at density of 3000 cells per well and 10 000 cells per well respectively. They were incubated for 24 h to adhere. Cells cultured in fresh medium served as blank control. Cell viability was acquired through cell counting kit‐8 (CCK‐8) assay using a Thermo Scientific Varioskan Flash microplate reader (Thermo, U.S.A) at day 1, 2, and 3 respectively. Three replicates were measured for each sample.

### Biocompatibility Experiments in Rats

200–250 g male rats were used (Chongqing, China, License No.: SYXK 2017‐0010). All animal experiments were approved by Laboratory Animal Welfare and Ethics Committee of Third Military Medical University (No. AMUWEC20223584). Dorsal skin was shaved and disinfected using 75% ethanol. Then three wounds were produced on both sides of the spine. Three sterilized‐P(3HB‐*co*‐4HB) sheet (80 × 80 × 0.4 mm) were implanted into the wounds randomly under sterile conditions. Following implantation of the test materials, wounds were covered with antibiotic cream (mupirocin). At predetermined time intervals (1, 3, and 6 months), the rats were euthanized and the skin specimens were dissected for histological staining.^[^ ^51^ ^]^

### The Incorporation of PEG with TA and In Vitro Inhibition of 3T3 Cells Experiment

Different concentrations of PEG (0%, 5%, 10%, and 15% m_PEG_/m_P(3HB‐_ *_co_*_‐4HB)_), TA (10% m_TA_/m_P(3HB‐_ *_co_*_‐4HB)_) and P(3HB‐*co*‐4HB) (12% m/v) were dissolved in chloroform and tubular structure was obtained like above. The structure was cut to 1 cm in length for compression experiment. Different concentrations of PEG (0%, 5%, 10%, and 15% m_PEG_/m_P(3HB‐_ *_co_*_‐4HB)_), TA (10% m_TA_/m_P(3HB‐_ *_co_*_‐4HB)_), and P(3HB‐*co*‐4HB) (12% m/v) were dissolved in chloroform and a film was acquired like above for tensile experiment.

The films in four groups (P(3HB‐*co*‐4HB), 5%PEG‐P(3HB‐*co*‐4HB), 10%PEG‐P(3HB‐*co*‐4HB), and 15%PEG‐P(3HB‐*co*‐4HB) were cut into rectangular thin sheet (1.2 cm × 0.5 cm × 0.1–0.15 mm). Each sheet was placed in an eppendorf tube with 1 mL PBS (pH = 7.4). The tube was placed on a shaker at 37 °C and the speed was set at 80 rpm. At different time points, the initial medium was discarded and 1 mL fresh PBS was used to replaced it. When determining the amount of released drugs, TA was extracted using choloroform first. The mixture was vortexed and centrifuged. The collected supernatant was then filtered with a 0.22 µm pore‐size filter and the concentrations were assessed by HPLC (methanol to water, 60:40 v/v; flow rate, 0.3 mL min^−1^; run time, 3.5 min; wavelength 240 nm; injection volume, 2 µL). Standards of TA were used for calibration. The test was conducted in triplicate and the results were presented as an average value.

For in vitro inhibition of drugs, 3T3 cells were cultured as above. Fresh medium with different concentrations of TA (0, 5, 10, 25, and 45 µg mL^−1^) were used to culture cells and replaced every day. The vitality of adherent 3T3 cells were evaluated by CCK‐8 at day 1, 2, and 3. There were six replicates for each sample among which three were used to study cell morphology. Cells were dyed to study cell morphology using Cell Viability Assay Kit according to instructions. Briefly, each well was added 100 µL dye buffer and then incubated darkly for 1 h at 37 °C. Fluorescence images were taken using an inverted fluorescence microscope (Olympus IX83, Japan) with excitation light of 488 nm.

### The Incorporation of EPSO

Different concentrations of EPSO (0%, 1%, 3%, 5%, and 10% v/v) and P(3HB‐*co*‐4HB) (12%, m/v) were dissolved in chloroform and a tubular structure was acquired like above. The stent was cut to 1 cm in length for compression experiment. The visibility of stent was evaluated under X‐ray (Philips Cardiovascular angiographic systems, 155–200 lb). Different concentrations of EPSO (0%, 1%, 3%, 5%, and 10% v/v) and P(3HB‐*co*‐4HB) (12%, m/v) were dissolved in chloroform to obtain a film like above for tensile experiment.

### In Vitro Degradation Study

The P(3HB‐*co*‐4HB) films (0%PEG‐P(3HB‐*co*‐4HB), 5%PEG‐P(3HB‐*co*‐4HB)) was cut into 0.8 cm × 0.8 cm × 0.2 mm and dipped in 2 mL PBS (pH = 7.4), minipig bile, and HCl (pH = 1) at 37 °C respectively with media replaced every week. At each time point, the specimens were taken out, rinsed with deionized water, wiped with absorbent paper and dried at 50 °C overnight. Then the mass would be recorded. Degradation was recorded as percent mass loss at each time point. As for the pH monitoring, the media in Eppendorf tube was not discarded. At predetermined time points, the pH change was estimated using pH dipstick.

### Stent Fabrication

The stent was divided into the main body with helical structure and tubular structures with teeth on both sides. The main body was built as follows. P(3HB‐*co*‐4HB) (12% m/v), PEG (5% m_PEG_/m_P(3HB‐_ *_co_*_‐4HB)_), EPSO (1% v/v), and TA(10% m_TA_/m_P(3HB‐_ *_co_*_‐4HB)_) were dissolved in chloroform and stirred thoroughly. Film was prepared by pouring mixed solution above on glass substrate with grooves and free dried. Then the film with outer track was cut into strips, twisted helically on metal rod with different diameters and fixed for 48 h to form a helical stent. As for the construction of the tubular structure with teeth at both ends. The mixed solution was poured into a glass tube with inner diameter of 2.2 mm, evaporated naturally, and was dried in oven to get a tubular stent. This stent was cut to 1.5 cm in length. A tooth was made out from the tube with knife carefully. P(3HB‐*co*‐4HB)(30% m/v), PEG (5% m_PEG_/m_P(3HB‐_ *_co_*_‐4HB)_), EPSO (1% v/v), and TA (10% m_TA_/m_P(3HB‐_ *_co_*_‐4HB)_) was dissolved in chloroform and stirred thoroughly. By applying solution on the contact surface, the main body of the stent was connected with the tubular structures. Finally, the surface of the stent was modified with a polishing machine and sterilized in a hydrogen peroxide low‐temperature plasma sterilizer.

### Mechanical Characteristics

The mechanical property of the stent was to test the main helical structure of the stent.

### Cyclic Compression Tests

Cyclic compression tests were performed on universal material testing machine (Sanfeng, Jiangsu)) with a 1000 N load cell using plastic stents and helical stents with different diameters and thickness (1 cm in length) at a speed of 10 mm min^−1^. Compressive strain was set at 25% and 50%.

### Cyclic Tensile Tests

Tensile cycling tests were performed on helical stents (1 cm in length) at a rate of 50 mm min^−1^. Tensile strain was set at 50%.

### Sludge Attachment Test

P(3HB‐*co*‐4HB) stents without PEG and TA were immersed in minipig bile and plastic stent with same surface area were used as control. The P(3HB‐*co*‐4HB) stent was unfolded to rectangle shape from helical shape. Samples were removed from the bile every 7 days and washed with distilled water to remove the loose biliary sludge on the surface. Then the mass and thickness of samples were recorded after they were dried to constant weight. The bile was replaced every 1 month.

### Investigation of shape recovery effect

P(3HB‐*co*‐4HB) films (thickness 0.2 mm) were made by pouring chloroform solution (5% w/v) on glass dish and dried to constant weight. P(3HB‐*co*‐4HB) films in strip (20 × 2 mm), round shape (11 × 11 mm), square shape (13 × 13 mm), flower shape, and star shape (solid and hollow) were made from film for test as mentioned above. The shape recovery behaviors of samples were investigated in two steps: 1) The P(3HB‐*co*‐4HB) films of different shapes were immersed in acetone to observe their shape changes. 2)After its shape was stable, the film was taken out and then put in air to observe shape changes. This process continues for three cycles. A digital camera was utilized to attain a continuous observation for the recovery process.

The shape memory of P(3HB‐*co*‐4HB) helical stents in acetone (room temperature), PBS (37 °C), bile (37 °C), and alcohol (37 °C) was tested in three steps: 1) The stent was placed in a glass tube of 1.3 mm inner diameter. 2) Different solvents were added to soak the stent and the change of the stent over time was observed. 3) After its shape was stable, the stent was removed and placed into another empty glass tube.

A wire was attached to one end of the stent and the stent was suspended in a conical flask. 50 mL acetone was added to the conical flask. The bottle was sealed with a Vinolok and the change of the stent over time was observed. When the shape stabilizes, the stent was removed from the bottle, put in the air, and the shape change was watched.

### Self‐Expanding Test

The P(3HB‐*co*‐4HB) stents with outer diameter of 3.2–3.5 mm and length of 8 mm were dipped in 2 mL PBS, bile, alcohol, acetone at 37 °C and room temperature. At predetermined time points, the stent was removed from tube and dried with absorbent paper. Then the outside diameters were measured.

The P(3HB‐*co*‐4HB) stent (3.2 mm in outer diameter and 1 cm in length) was placed in a big glass disc. The self‐expanding force was measured by a universal testing machine after the acetone was added into the disc and submerged the stent completely.

### Animal Preparation and Grouping

All the animal experiments conformed to the ethical guidelines and obtained permission from the Laboratory Animal Welfare and Ethics Committee of Third Military Medical University (Chongqing, China, License No.: SYXK 2017‐0002). Minipigs were bought from local farms with average weight of 15–25 kg. Animals had free access to water and food and were raised in single cage under conditions of 22 °C, 55% humidity with a 12‐h diurnal rhythm. Minipig were fasting and allowed to drink glucose saline only 2 days before the experiment. The minipig were anesthetized using xylazine hydrochloride by intramuscular injection (HuaMu animal health products co. Ltd. Jilin, China.) and maintained by intravenous injection of propofol (LIBANG Pharmaceutical Co. Ltd. Xi' an, China).^[^ ^52^ ^]^ During anesthesia, oxygen was given through a nasal tube at a rate of 2 L min^−1^. Four minipigs were used for experiment successively. Minipig no.1(normal group) and no.2 (stent group) were not modeled and the bile duct was normal. A helical stent without TA was inserted into bile duct of no.2 minipig. Minipig no.3 (model group) and no.4 (TA‐stent group) were modeled with biliary stricture. A helical stent loading TA with teeth at both ends was inserted into no.4 minipig.

### Modeling of Biliary Stricture in Minipig

ERCP was performed by expert endoscopist using electronic colonic endoscopy (CF‐HQ290I; Olympus, Japan) to obtain a cholangiogram (Figure 2A). Then, Cystotome Cystoenterostomy Needle Knife (CST‐10, Cook Medical Trading Co. LTD) was inserted. The knife was advanced into the distal common bile duct (CBD) 2 cm from the duodenal papilla over the guide wire under fluoroscopic guidance (Figure 2C). Electrical cauterization was performed intermittently at 30 W power for a total of 4 s. After the operation, ceftriaxone (1 g day^−1^) was intraperitoneally injected for 3 days to prevent biliary tract infection. Blood was taken for test every 5 days until 20 days after the operation. The sclera color was observed. When the blood test index related to obstruction of biliary tract increased obviously and sclera color turned yellow, ERCP and choledochoscope (EyeMax Insight ERC Imaging system, Nanjing Minimally Invasive Medical Technology Co., LTD) were used to evaluate the success of the model.

### Stent Placement

When stricture formed, a stent was placed into the bile duct under endoscopy after endoscopic biliary sphincterotomy. Blood samples were taken at predetermined time point for further test and the sclera color was observed. X‐ray was used to observe the position and shape of the stent. After endoscopic procedure, all animals were allowed to eat their regular diet and animals were euthanized by infusing an overdose of xylazine hydrochloride finally.

### Blood Analysis

Blood was collected at predetermined time point for routine blood test and biochemical test (TBIL, DBIL, γ‐GT, ALT, AST, LDH, IL‐6, TNF‐α, IL‐8).

### Histological Evaluation

No. 1 and no. 2 minipigs were euthanized after 1 month of stent implantation in no. 2 minipig. No. 3 minipig were euthanized after modeling for 20 days. No. 4 minipig was euthanized after stent implantation for 2 months. The CBD containing stent, a section of liver, and duodenum were removed. The bile duct was fixed in 10% formalin for at least 24 h. H&E, Masson staining, and α‐smooth muscle actin (α‐SMA), CD31, PCNA immunohistochemical staining were performed.

Digital images of the slides were obtained using a digital pathology section scanner (HS6, Shunyu, Ningbo). Four fields of view were used to analyze and quantify for each sample. The assessment indicators in HE included severity of mucosal sloughing, atrophy inflammation, fibrosis, and hyperplasia. The degree of submucosal inflammation was classified as 0 (none), 1 (mild), 2 (moderate), or 3 (severe). Collagen deposition was evaluated in Masson staining. α‐SMA immunohistochemical staining was used to show positive cells which included myofibroblasts and vascular smooth muscle cells which served as positive controls. PCNA immunohistochemical staining was used to estimate proliferation of tissue. Semiquantitative analysis was performed by Image J.

### SEM of Stent Removed from Biliary Duct

At predetermined intervals, stents were removed and imaged on a scanning electron microscope (Crossbeam 340, Zeiss).

### Statistical Analysis

All data were shown as mean ± SD to evaluate differences. Statistical analysis was carried out by independent‐samples T test, one‐way analysis of variance, and nonparametric rank‐sum test using Graphpad 8.0.1 software. Statistical significance was considered when *P* < 0.05. * indicates *P* < 0.05, ** indicates *P* < 0.01, *** indicates *P* < 0.001.

## Conflict of Interest

The authors declare no conflict of interest.

## Supporting information

Supporting Information

Supplemental Video 1

Supplemental Video 2

Supplemental Video 3

Supplemental Video 4

Supplemental Video 5

Supplemental Video 6

Supplemental Video 7

Supplemental Video 8

## Data Availability

The data that support the findings of this study are available from the corresponding author upon reasonable request.
