# Supplementary material for: Biosynthetic Plastics as Tunable Elastic and Visible Stent with Shape‐Memory to Treat Biliary Stricture
Source: Adv Sci (Weinh). 2023 Aug 8;10(29):2303779. doi: 10.1002/advs.202303779 (PMC10582434; doi:10.1002/advs.202303779)
Supplement: Supplementary file 1 — Supporting Information [file ADVS-10-2303779-s002.pdf]

ADVANCED SCIENCE Open Access

Supporting Information

for *Adv. Sci.*, DOI 10.1002/advs.202303779

Biosynthetic Plastics as Tunable Elastic and Visible Stent with Shape-Memory to Treat Biliary Stricture

Wei Wang, Zhaohui Luan, Zhenzhen Shu, Kaige Xu, Tongchuan Wang, Shuang Liu, Xiaozhuo Wu, Hangzong Liu, Shaosong Ye, Ruijue Dan, Xiaoyan Zhao, Shiming Yang*, Malcolm Xing* and Chaoqiang Fan**

Supporting Information

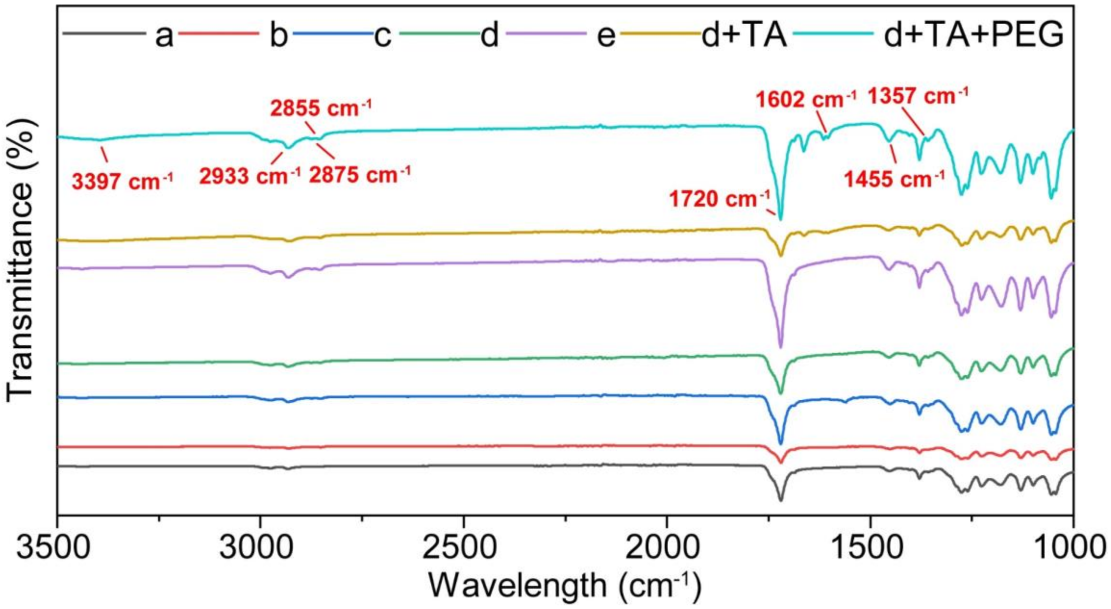

Figure S1. Five materials (a-e) and the mixture of d with TA and PEG were characterized by FTIR.

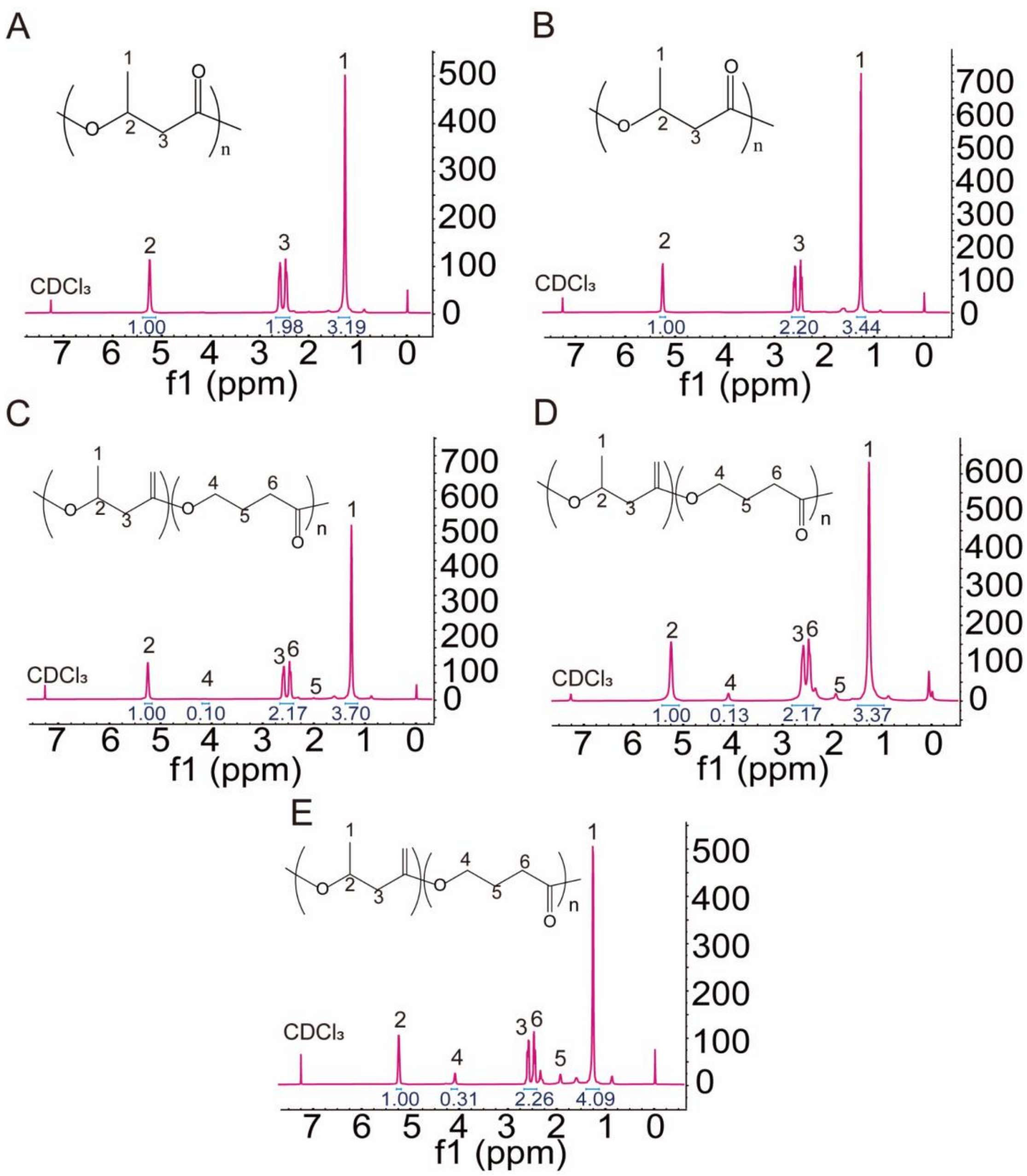

Figure S2. A-E: Five materials (a-e) were characterized by ^{1}HNMR.

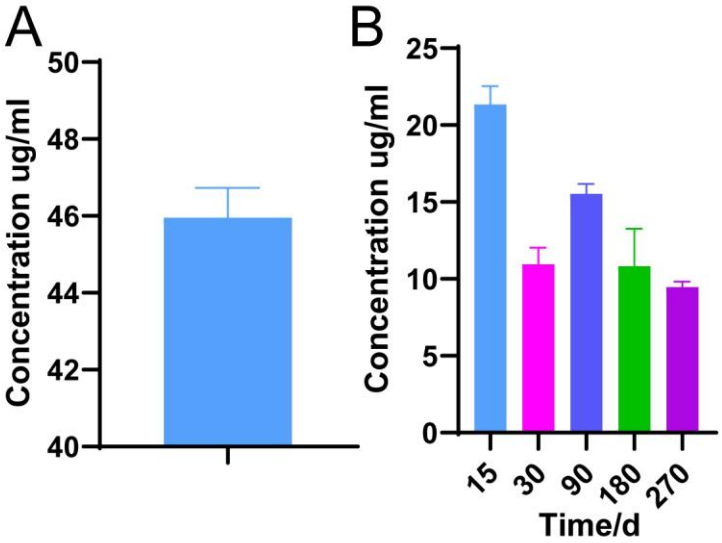

Figure S3. The release profile of TA. A: The maximum concentration of TA in DMEM medium. B: Concentrations of drug released by TA-loaded P(3HB-co-4HB) film at different time points.

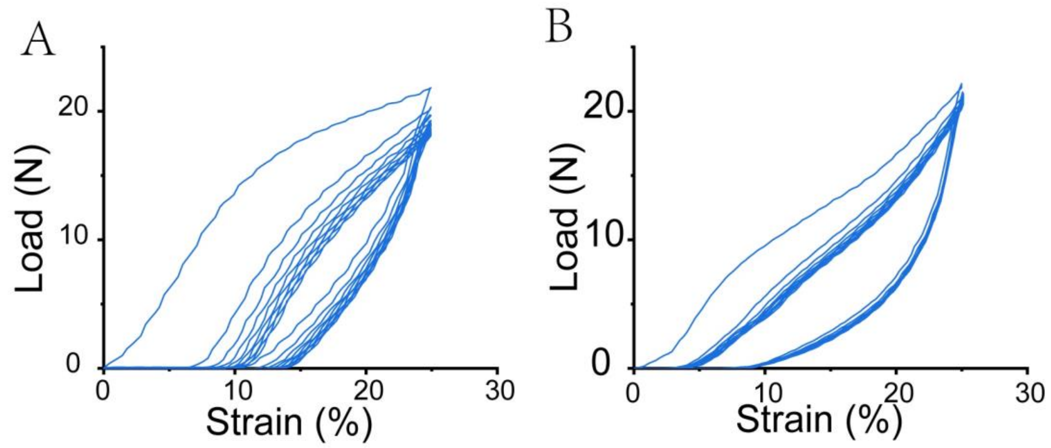

Figure S4. Cyclic compression test of the plastic stent and P(3HB-co-4HB) stent. A: The cyclic compression performance of plastic stent (strain 25%). B: The cyclic compression performance of P(3HB-co-4HB) stent (2.8mm in diameter, 0.4mm in thickness, strain 25%)

A B

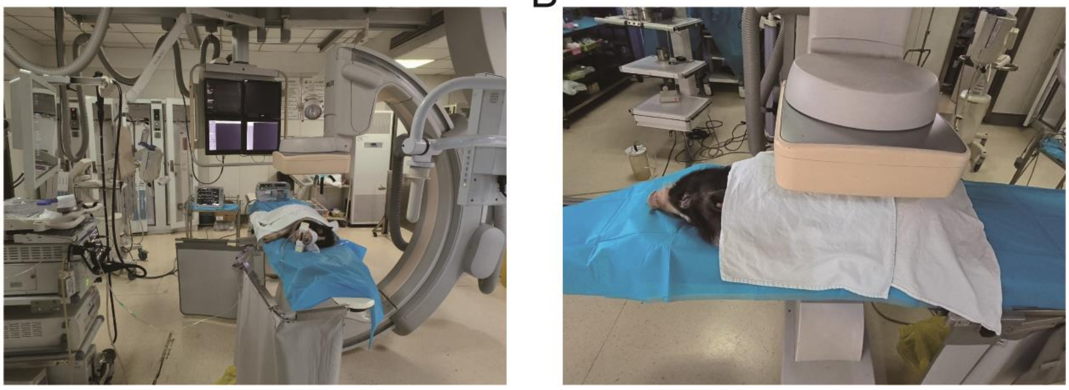

Figure S5. Scene of operation on minipigs endoscopically and observation under x-ray.

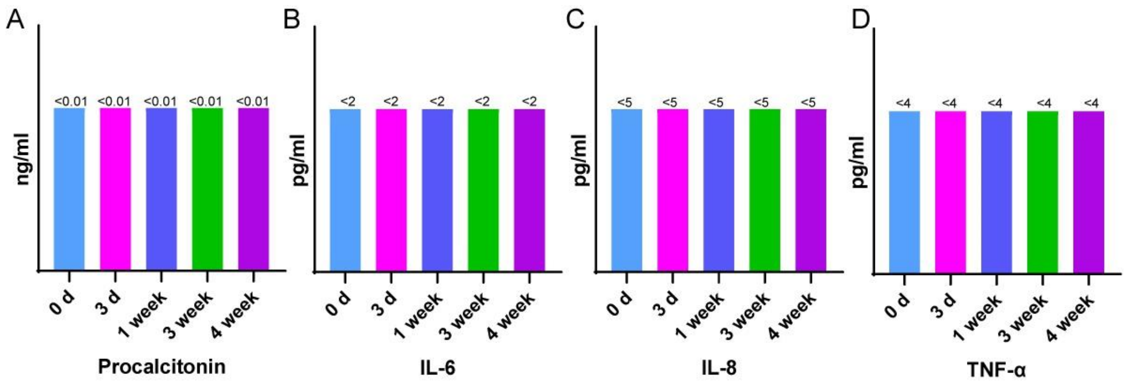

Figure S6. Changes of serum procalcitonin and some inflammatory factors in No.2 minipig at different time points. The index detection was below the detection range, indicating no obvious inflammation.

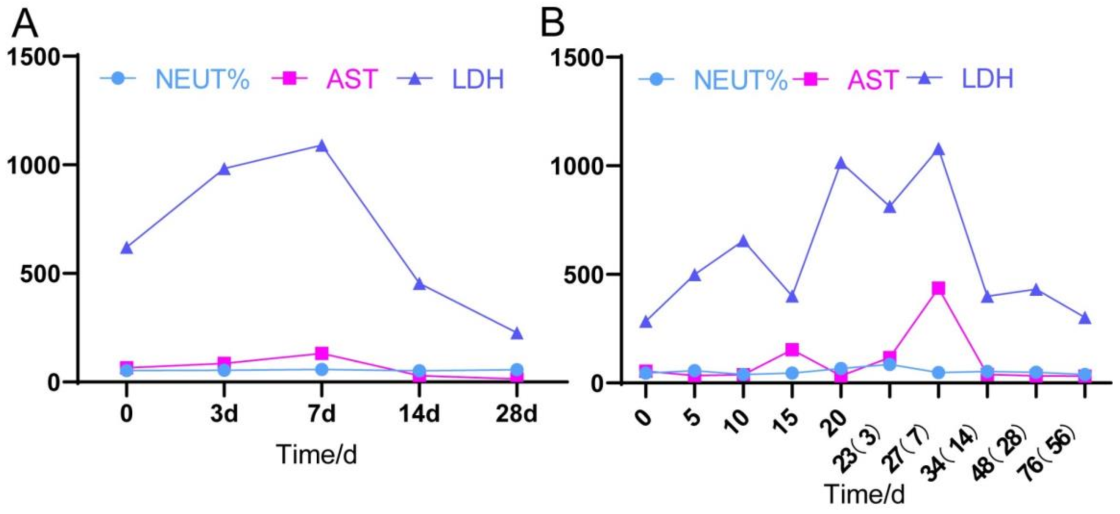

Figure S7. Changes of percentage of neutrophils, AST and LDH in No.2 and No.4 minipigs. AST: aspartate aminotransferase, LDH: lactic dehydrogenase and NEUT%: neutrophilic granulocyte percentage. Days after stent placement were shown in parentheses.
